# Effectiveness of Virtual Reality for Children and Adolescents with Autism Spectrum Disorder: An Evidence-Based Systematic Review

**DOI:** 10.3390/s18082486

**Published:** 2018-08-01

**Authors:** Patricia Mesa-Gresa, Hermenegildo Gil-Gómez, José-Antonio Lozano-Quilis, José-Antonio Gil-Gómez

**Affiliations:** 1 Departamento de Psicobiología, Facultad de Psicología, Universitat de València, Blasco Ibáñez 21, 46010 Valencia, Spain; patricia.mesa@uv.es; 2 Instituto Universitario de Automática e Informática Industrial, Universitat Politècnica de València, Camino de Vera s/n, 46022 Valencia, Spain; hgil@ai2.upv.es (H.G.-G.); jlozano@upv.es (J.-A.L.-Q.)

**Keywords:** virtual reality, ASD, Autism Spectrum Disorder, augmented reality, Asperger

## Abstract

Autism Spectrum Disorder (ASD) is a neurodevelopmental disease that is specially characterized by impairments in social communication and social skills. ASD has a high prevalence in children, affecting 1 in 160 subjects. Virtual reality (VR) has emerged as an effective tool for intervention in the health field. Different recent papers have reviewed the VR-based treatments in ASD, but they have an important limitation because they only use clinical databases and do not include important technical indexes such as the Web of Science index or the Scimago Journal & Country Rank. To our knowledge, this is the first contribution that has carried out an evidence-based systematic review including both clinical and technical databases about the effectiveness of VR-based intervention in ASD. The initial search identified a total of 450 records. After the exclusion of the papers that are not studies, duplicated articles, and the screening of the abstract and full text, 31 articles met the PICO (Population, Intervention, Comparison and Outcomes) criteria and were selected for analysis. The studies examined suggest moderate evidence about the effectiveness of VR-based treatments in ASD. VR can add many advantages to the treatment of ASD symptomatology, but it is necessary to develop consistent validations in future studies to state that VR can effectively complement the traditional treatments.

## 1. Introduction

Autism Spectrum Disorder (ASD) is an atypical neurodevelopmental disease characterized by impairments in social communication, interaction, competences, and language, as well as the maintenance of restricted and repetitive behaviors, interests, and activities [1,2]. Clinical heterogeneity of this disease is well known, and the children affected show other symptomatology such as hyperactivity or lack of attention that could be related to attention deficit hyperactivity disorder (ADHD) [3]. 

From its first known use in 1987, many different definitions for virtual reality (VR) are available in the literature. An interesting recent definition [4] defines VR as “an artificial environment which is experienced through sensory stimuli (such as sights and sounds) provided by a computer and in which one’s actions partially determine what happens in the environment”. In a broader sense, VR includes some interactive video gaming, virtual environments, and, commonly, a multisensory experience. VR uses many different technologies: monoscopic or stereoscopic displays, user tracking technologies, augmented reality (AR) to merge real and virtual worlds, etc.

VR has emerged as an effective new treatment approach in different areas of the health field, such as rehabilitation [5,6], promotion of emotional wellbeing in inpatients [7,8], diagnosis [9,10], surgery training [11,12] and mental health treatment. With regard to mental health treatment, VR is used in the treatment of a wide range of disorders: Phobias, post-traumatic stress disorders, obsessive-compulsive disorders, and, of course, ASD. Specifically, in this intervention area, VR has shown some advantages, allowing ASD patients to be trained in a realistic environment that could be manipulated and adapted to the characteristics and capabilities of the subject. It has been related to the ecological validity of treatments of this type in a controlled environment [13].

In the last few years (since 2015), previous reviews concerning ASD and VR have made some contributions to the exploration of these topics. Mishkind et al. [14] provide a review of VR treatment in psychiatry. However, this review is specially focused on other disorders (i.e., post-traumatic stress disorder, anxiety and phobias, chronic pain, rehabilitation, and addictions), and they do not review most of the recent studies concerning ASD and VR. In 2017, Liu et al. [15] published a comprehensive review of technology-facilitated diagnosis and treatment of ASD. This study is different to our study in many aspects, but the main difference is that they focus their review on the engineering perspective of autism studies. In 2017, van Bennekom et al. [16] carried out a literature review in which they evaluated the assessment of psychiatric disorders by means of a VR environment. However, they were not focused on ASD and they excluded studies if VR was used for therapeutic purposes.

A systematic review was published in 2017 by Provoost et al. [17]. In this contribution, the authors provide an overview of embodied conversational agents (ECAs) for the delivery of automated human support factors. ECAs are computer-generated characters that are used for human face-to-face conversation simulations. Thus, this review does not cover studies that involve VR if they do not also include ECAs. Lau et al. [18] published a systematic review that partially matches the aim of this contribution. The main difference is that their review analyzes the use of serious games for mental health disorders. However, some serious games cannot be considered VR systems, and, also, some VR systems cannot be considered serious games. Sarah Parsons contributed a very interesting review [19], but it was not systematic. Instead, she carried out a conceptual review, raising questions about the assumption of veridicality of VR for autism. In 2015, den Brok and Sterkenburg [20] contributed a systematic review that examined the studies that applied self-controlled technologies to support skill attainment. However, they did not consider some interesting studies concerning VR and ASD, and they included other studies that focused on people with intellectual disability.

Our contribution also has an important added value: Most of the systematic reviews have carried out a comprehensive literature search in PubMed, Embase, and/or Psycinfo. These indexes are focused on clinical and biomedical literature. In our contribution, we include clinical indexes, but we also include the Web of Science (WoS) index and the Scimago Journal & Country Rank (SJR) from the Scopus database. WoS and SJR are interdisciplinary indexes that include contributions from different areas. With all of these indexes, our contribution covers a wider range of publications, offering a very complete review.

## 2. Materials and Methods

### 2.1. Search Strategy

The systematic review performed a comprehensive literature search of PubMed, WoS, and SJR. PubMed is a free resource that is maintained by the National Center for Biotechnology Information. PubMed includes more than 28 million citations for biomedical literature from MEDLINE, life science journals, and books. WoS is a scientific citation indexing service that was produced by the Institute for Scientific Information and is maintained by Clarivate Analytics. WoS comprises more than 1.4 billion citations, providing thorough coverage and comprehensive indexing of the journals, books, and proceedings in the sciences, social sciences, and arts and humanities. SJR is a publicly available portal that includes the journals and country scientific indicators developed from the information contained in the Scopus^®^ database (Elsevier B.V., Amsterdam, The Netherlands).

Since technology is an essential part of the studies involving VR and the advancement in technology has been exceptionally fast in this century, only recent articles (from 1 January 2010 to 28 February 2018) were considered in the systematic review. The search terms were: (autism OR ASD OR Asperger) AND (Virtual OR VR OR Mixed OR Augmented). Furthermore, the articles in the lists of reviews described in the introduction section [14,15,16,17,18,19,20] were searched manually.

Contributions of the following types were not considered: Reviews, letters, abstracts, editorials, and notes. We only included studies where the authors carried out the evaluation of the impact of a VR-based treatment in children with ASD. Only studies in English were considered during the search.

### 2.2. Selection Criteria

Defining the correct question in the selection criteria is critical in finding clinically relevant evidence in the literature. Thus, we selected the PICO model for this purpose because a good question should include four parts that identify the patient problem or population (P), intervention (I), comparison (C), and outcome(s) (O). The following PICO question was established for the literature selection procedure:

- P—Children (age < 18) with ASD
- I—VR-based treatment
- C—(versus) non-VR-based treatment, children’s condition before VR-based treatment, without treatment.
- O—Main outcomes obtained, no significant improvement is needed.

Because of the nature of research and technological limitations, VR systems are not yet widely used as clinical interventions. Most of the studies are short-time-exposure pilot studies, a limitation that is especially relevant in social interaction behavioral intervention. Since significant outcomes in literature are not expected, we used the PICO model but relaxed the emphasis on outcome (O).

### 2.3. Selection Process

First, a search with the terms (autism OR ASD OR Asperger) AND (Virtual OR VR OR Mixed OR Augmented) was done in each index (WoS, SJR, and PubMed). In the PubMed search, only studies that focused on humans were selected. Second, documents belonging to the categories of reviews, letters, abstracts, editorials, and notes were discarded from the results obtained in each index. Third, from the rest of the results, we carried out a selection of the contributions based on titles and abstracts in each index; then the results of the selection in the three indexes were compared to eliminate duplicates.

Last, the full text of each article was examined, and the contribution was finally selected if: (1) The study included participants with ASD under 18 years old; (2) the intervention employed VR-based treatments; (3) baseline or intergroup comparisons were done; and (4) outcomes on patients were reported. We resolved any disagreements in the selection of the articles among the authors of this work.

### 2.4. Data Extraction

For each study, we summarized the following information: Diagnosis of the participants; sample size of the groups, experimental group and control group (if any); age range and sex of the participants (in each group); aim of the study (enhance emotional skills, improve communication ability, etc.); main technology used for both hardware (head-mounted displays, mobile devices, etc.) and software (augmented reality, VR driving simulator, etc.); methodology (sessions/weeks, duration of the sessions, etc.); evaluation (when the evaluation was done and tests/scores used); and results (improvements and significance).

## 3. Results

### 3.1. Study Selection

**Figure 1:**
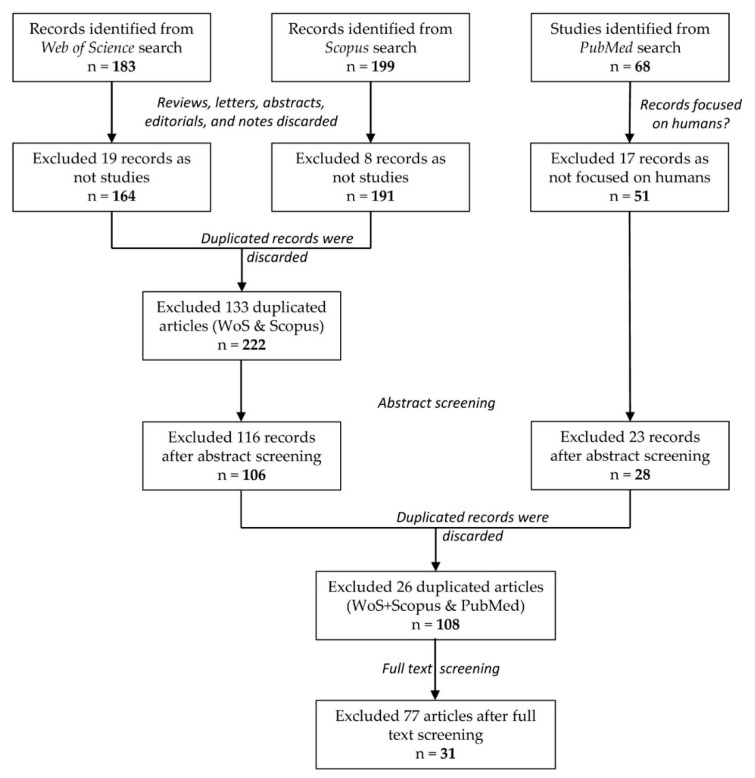
Flow diagram showing the overview of the search/selection process. The initial search in the three indexes identified a total of 450 records. After the exclusion of the papers that are not studies, the exclusion of the duplicated articles, and the abstract screening, we carried out a full text screening of 108 papers. Finally, 31 articles met our criteria and were selected for analysis.

A flow diagram showing the overview of the search/selection process is depicted in Figure 1.

The search in the selected indexes selected 450 potentially relevant contributions: 183 records in WoS, 199 records in SJR, and 68 records in PubMed. For the WoS and SJR indexes, 19 records from WoS and 9 records from SJR were discarded because they were reviews, letters, abstracts, editorials, or notes. The remaining 355 papers from WoS & SJR were examined to eliminate duplicates: 133 duplicated articles were excluded. The abstract screening of the 222 remaining articles resulted in the selection of 106 studies as candidates to fulfill the inclusion criteria.

For the PubMed index, 17 of the 68 initial records did not focus on humans. Of the remaining 51 records, an abstract screening excluded 23, leaving 28 papers after this step. After the inspection of the set of papers remaining in WoS+SJR (106 records) and the set of papers remaining in PubMed (28 records), 26 duplicated contributions were discarded. We only considered the papers that were exactly the same article to be duplicated contributions (i.e., the same title, the same authors, and the same publication). Finally, 108 articles were read in order to select the contributions that met all of the criteria. A total of 31 records fulfilled the criteria and were included in the data extraction that is shown in Table 1.

### 3.2. Study Characteristics

A total of 602 subjects participated in the 31 selected papers; 451 participants were in an Experimental Group (EG) and 151 participants were in a Control Group (CG). Only ten studies compared EG vs CG, while 21 studies evaluated only the impact of the intervention in an EG. For the ASD participants who were in an EG in studies where the distribution males/females was specified, 85.15% of the subjects were males and 14.85% were females.

**Figure 2:**
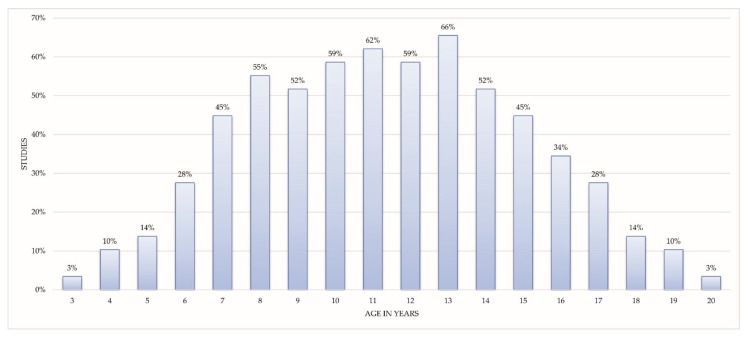
Percentage of studies that included participants of a specific age.

Twenty-nine studies specified the age range of the participants, while only two studies indicated that the participants were children. The youngest participant was 3 years old, and the oldest was 20 years old. The mean age of the subjects in the different studies ranged from 5 to 15.5 years old. Of these 29 studies, the percentage of studies that included subjects of a specific age is shown in Figure 2. The data show that at least half of the studies involved children in the range of 8–14 years old.

Four studies (12.90%) included children diagnosed with Asperger, and six other studies (19.36%) included high-functioning ASD children. Only one study focused on low-functioning ASD children. In addition, one study focused on ASD children with attention deficit hyperactivity disorder. Last, there was one study that included children suspected of having ASD in the EG.

With regard to the effectiveness of the studies, 30 of the 31 studies stated that the application of the VR-based treatment resulted in the improvement of at least one of the objectives addressed. Only one study specified that the results were inconclusive. However, only 10 of the studies identified the improvement as being statistically significant.

**Figure 3:**
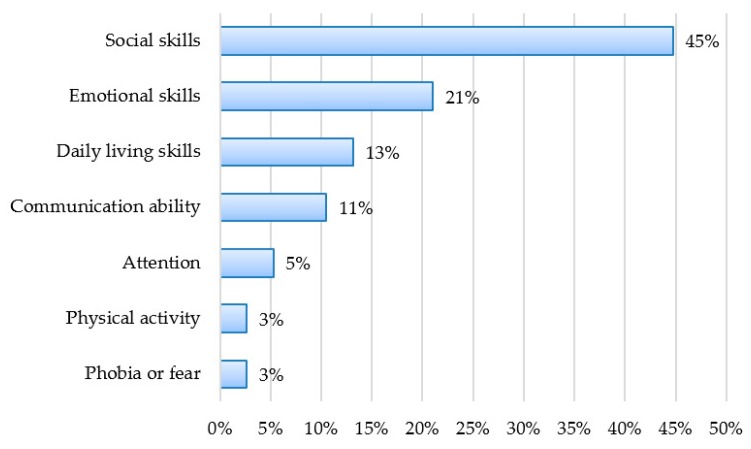
The percentage of studies that included each clinical target.

The clinical focus of most of the studies was on emotional and/or social skills (55.26%), including emotion recognition, collaboration, and social interaction tasks. A total of five studies (13.16%) try to improve daily living skills, especially shopping and driving. Four studies (10.53%) developed the communication ability of children. Two studies (5.26%) were focused on attention. Only one study (2.63%) was targeted to improve physical activity, and one study (2.63%) was designed to reduce a specific phobia or fear. Figure 3 shows the percentage of studies that focused on each clinical target (note that some studies focused on two or even three targets).

If we specifically consider the clinical target of the studies reviewed, we can observe some interesting data, which are described in the following sub-sections.

#### 3.2.1. Social Skills

Seventeen manuscripts of the 31 selected addressed the intervention of social skills in children with ASD [21,22,26,28,29,32,34,36,37,38,42,43,44,46,47,50,51]. The age range on which the intervention was performed was 4 to 17 years old, with the average age being 10.5 years old. In the papers analyzed, a total of 142 boys and 22 girls participated. The main technologies used include virtual reality scenarios, in which collaborative tasks or scenarios based on a second life games can be carried out.

#### 3.2.2. Emotional Skills

Eight articles were obtained for this area of intervention. The age range was 4 to 19 years old with the average age being 10 years old [21,22,27,29,30,33,35,48]. The total samples were composed of 74 boys and 20 girls. The type of technology most commonly used was VR scenarios and environments, in some cases with an avatar. One study was observed using technology based on a collaborative virtual environment (CVE). In studies in which the main objective was to identify core emotions (two studies), augmented reality scenarios were employed.

#### 3.2.3. Daily Living Skills

Interventions of this type are based on the training of the subjects in tasks related to daily living, such as driving or shopping in a supermarket. Five studies were selected for the present review [24,25,31,47,49]. The age range was 6–19 years old and the average age was 12.5 years old. The samples included were composed of 67 boys and 10 girls. The main technology used in these studies included VR-scenarios and VR-driving modules.

#### 3.2.4. Communication Ability

Four articles are included in this category [23,36,44,45]. Only two of the manuscripts selected included a complete description of the samples, with an age range of 6–17 years old with the average age being 10.75 years old. Only one of the articles specified the gender of the participants, with 13 boys and 2 girls. The technology used includes AR and VR elements, CVE games, and Ms Kinect.

#### 3.2.5. Attention

Two manuscripts based on the training of attention were selected [22,39]. These studies present samples with an age range of 3–8 years old (average age of 5.25 years old). The gender of the participants was not specified. The technology used is based on VR-scenarios and the Mobile Object Identification System.

#### 3.2.6. Physical Activity

One manuscript [40], which aimed at increasing motivation for physical activity, has been included in this review (age range 8–20 years old, with an average age of 14 years old). Immersive stereoscopic surround-screen, BodyMedia, and SenseW armband were used as technological devices.

#### 3.2.7. Phobia or Fear

Only one study was conducted to include technology in the psychological intervention of phobia or fear in children with ASD [41], with an age range of 7–13 years old and an average age of 10 years old. This study was carried out with a sample of all boys. The technology used included Blue Room Virtual Reality Environment (VRE) with individualized scenes.

## 4. Discussion

The main aim of this systematic review was to evaluate and describe the main results that are related to the effectiveness of the application of VR programs in the intervention and treatment of children and/or adolescents with Autism Spectrum Disorder. Thirty-one research papers (published between 2010–2018 and obtained from the Pubmed, WoS and Scopus databases) were selected and analyzed for this review.

With regard to the characteristics of the participants, the mean age of the subjects enrolled in the studies ranged from 5 to 15.5 years old. However, the age range of 8–14 years old was the main focus of the studies (only two studies did not include children in this range). As expected for ASD children, the participants were mainly boys, and the ratio of boys to girls observed in the studies was 4:1 (85.15% of the sample). This should be taken into account in the interpretation and extrapolation of the results, in contrast to the 3:1 ratio established by Loomes et al. [52].

In terms of areas of intervention and taking into account the possibilities offered by VR technologies, most of the studies are related to the improvement of activities in daily life and communication, especially social and emotional skills. Specifically, if the area of intervention is taken into account, we can distinguish six clear areas: Social skills, emotions, daily living activities (driving, shopping, etc.), communication, cognitive training, and other areas such as improvement of physical activity or motivation. 

Social skills, the main hallmark deficit in children with ASD, have received the most attention in the VR studies reviewed. As can be seen, a significant proportion of the studies analyzed have been based on this area of intervention (44.74%), since the use of avatars and virtual environments representing social situations allows the training in a safe and controlled environment that could be personalized. The features of this type of intervention are especially interesting for ASD therapy. From the studies reviewed, based on the exposure of subjects to social situations in virtual scenarios [26,28,29,32,34,35,37,38,42,43,46,47,50,51], the main results indicate important changes to be considered. In studies based on the use of second life games, improvements in emotional regulation, social attribution, and analogical reasoning were observed [28,29]. In the case of collaborative VR scenarios, results have indicated that the use of technology facilitates the training of flexibility, identity, the construction of social norms, and emotional recognition [32,35]. In addition, these strategies contribute to the development of important aspects related to social skills: Initiation of play, social response, and conversational skills. Avatars and virtual elements contribute to the training in recognition of facial expressions and body gestures [37,42,46]. Moreover, virtual environments permit the recreation of specific situations, such as school [47] or bullying [51].

The articles that focus on the intervention on emotions [21,22,27,29,30,39,48,51] are mainly based on the use of avatars or games for training and learning aspects such as the identification of basic emotions [27,33], the regulation of emotional expression, and social emotional reciprocity [21,22,29]. Some studies that focus on this intervention area have shown interesting results through virtual scenes that allow training in emotional recognition, affective expression, emotional competencies, and positive emotions [29,30,39,48].

Children with ASD usually have difficulty with communication skills. In the studies analyzed, the use of VR as an intervention in communication skills, social communication strategies, and non-verbal communication has been observed [23,44,45].

Other studies have been based on areas related to training in activities of daily living or cognitive skills. In the intervention on cognitive abilities, positive results have been obtained from the use of VR in attention [22,39], executive functions [47], contextual processing of objects, and cognitive flexibility [49]. This latter study [49] is especially interesting because one of the characteristics of some of the subjects suffering from ASD is cognitive rigidity. On the other hand, the use of intervention strategies based on VR or AR allows the training of the subject in virtual situations that are safe and controlled environments. An example of this is the use of VR to teach driving skills [31] or how to deal with a daily situation such as shopping in a supermarket [24,25]. VR has also been used in combination with traditional therapies, such as cognitive behavioral therapy, to improve symptoms related to specific phobias or fears [41] and to motivate children with ASD to improve their lifestyle habits by increasing physical activity [40].

With regard to technology, most of the VR-based treatment included VR scenarios, whereas some of them only included objects and/or avatars that were not in a VR scenario. Augmented reality has recently been considered in the treatment, and four studies (all from 2015) used this technology. In addition, five studies used mobile devices in the intervention. From our point of view, these devices represent a step forward in the treatment of patients with ASD because they allow an approach that is ubiquitous to be used for autistic disorders. The main results appear to show positive outcomes from exposure to VR-based intervention in children with ASD. However, it is important to consider that the main results obtained involved small samples. Also, in most cases, there was no comparison with a control group composed of healthy volunteers or patients receiving traditional interventions. 

With regard to the symptomatology of ASD, VR-based treatments may have some advantages over more traditional interventions. One of the main advantages is that VR allows the emulation of everyday life situations so that scheduled training can be conducted in a therapist-controlled and safe environment. This issue is especially interesting when the treatment should be focused on the training and improvement of social skills, social interaction, communication, emotional response, or executive functions. In addition, this type of intervention can be developed further to obtain different measures of the performance of the subjects. This allows therapists to follow up and analyze the patient’s improvements and apply feedback or possible repetitions of the tasks. Thus, intervention based on technology could include multi-user applications in playful environments or everyday situations, which could be controlled and personalized based on the objectives of the intervention [53]. Previous studies applied to the rehabilitation of cognitive impairments have indicated that the VR interventions have high ecological validity and the learning acquired could be transferred to real life [54,55]. Another advantage is that VR includes the possibility of modifying and personalizing the tasks, measures, difficulty, situations, and stimuli included in the environment. Even the characteristics of the avatars in the environments can be personalized. Another positive contribution is the type of technology used: Most of the studies involve low-cost technology (e.g., Nintendo) allowing users to continue with a home-based treatment or permitting access to people who are unable to move around [56]. This could be interesting for treatment as well as for the possible improvement in situations of informal caregivers using technology in therapy. Since autism is diagnosed in children and many of the interventions are performed during adolescence and youth, the use of VR-based technologies as part of the treatment can increase the motivation and adherence of patients as well as their enthusiasm and implication in the therapeutic program.

It is important to consider, based on the main results obtained in this systematic review and the main results obtained in previous research, the limitations in the use of new technologies in the treatment of autism. The evidence of efficacy of VR-based treatment is limited. Many of the studies analyzed did not include control groups composed of subjects diagnosed with ASD that received other traditional therapies in order to compare differential effects of exposure to VR. Some studies included a control group composed of healthy children and other studies did not include control groups of any kind. The comparison of the changes obtained by a VR intervention could be difficult to determine if the control group did not show the same characteristics or if the same questionnaires were collected at different moments in the intervention program. Taking this into account, some results obtained by the studies reviewed here could be considered preliminary and limited for clinical practice. Another limitation of the studies is the number of subjects in the samples. The difficulty of obtaining large samples when the aim of the study is related to psychopathology is well known. The reviewed papers had small samples (only 4 studies—12.90%—included more than 30 subjects in the experimental group). Therefore, it is difficult to extrapolate the results to the population affected by this disease. Another issue that makes it difficult to extrapolate the results is the gender ratio of the sample. It is known that ASD affects more boys than girls (the updated literature establishes a 3:1 ratio [52]). However, some studies are conducted only with affected boys and this may be limiting the conclusions drawn. Finally, it should be noted that some studies were carried out on children diagnosed with high-performance autism or Asperger Syndrome. Thus, results should only be considered for this subsample as they could not be applied to the rest of the children with ASD.

## 5. Conclusions

For the studies analyzed, there is moderate evidence that VR-based treatments can help children with ASD. The lack of definitive findings does not allow us to state that VR-based treatments can improve the results of traditional treatments. Nevertheless, the promising results and the advantages of VR (especially considering ASD symptomatology) should encourage the scientific community to develop new VR-based treatments. Future studies must be validated through well-designed evaluation processes. The main limitations observed in the evaluation processes of the studies analyzed need to be improved. It could be interesting to consider expanding the samples and to include a boy-girl ratio of around 3:1. In addition, new areas of study could be added, such as training and evaluation of non-verbal communication or executive tasks applied to everyday situations. These are areas that have been poorly treated in the studies. The application of these interventions from home combined with the training of caregivers may also be of interest. This would strengthen the learning obtained during therapy and help to improve the interaction of patients with their caregivers, reducing the overload and stress suffered by them.

## Figures and Tables

**Table 1:** Summary of selected contributions (*n* = 31).

Contribution	Diagnosis	Groups (Sex)/Age	Aim	Evaluation	Main Results
Ip et al. [21] 2018	ASD	EG: 36 (31 M, 5 F) CG: 36 (33 M, 3 F) Age: 7–10 y/o	Enhance emotional and social adaptation skills.	FT, ET, PEP-3, ABAS-II.	Improvements in children’s emotion expression and regulation and social-emotional reciprocity.
Manju et al. [22] 2018	ASD	EG: 5 CG: - Age: 4–6 y/o	Enhance social skills, emotions and attention.	Specific scoring criteria based on a Likert scale method.	Improvements in all the participants, but statistical significance is not analyzed.
Taryadi and Kurniawan [23] 2018	ASD	EG: 12 CG: - Age not specified	Improve communication ability.	Subjective qualitative analysis.	Improvement of communication ability, but statistical significance is not analyzed.
Adjorlu et al. [24] 2017	ASD	EG: 4 CG: 5 Age: 12–15 y/o	Development of daily living skills (shopping skills)	Task completion time and effectiveness, questionnaires, observations.	Some of the indicators show significant improvement.
Lamash et al. [25] 2017	ASD	EG: 33 (29 M, 4 F) CG: 23 (17 M, 6 F) Age: 11–19 y/o	Improve the implementation of a shopping task.	WebNeuro, BRIEFSR, and the TOGSS.	Significant improvement of the EG compared to the CG in several indices.
Bekele et al. [26] 2016	HFASD	EG: 6 (6 M) CG: 6 (6 M) Age: 13–17 y/o	Emotion recognition in a social context.	Isolated emotion recognition test, NEPSY test.	System useful in training core deficit areas for eventual better social functioning.
Chen et al. [27] 2016	ASD	EG: 6 (5 M, 1 F) CG: - Age: 11–13 y/o	Identify the 6 core emotions.	Specific questions.	All scores rose significantly during the intervention and remained significantly high.
Didehbani et al. [28] 2016	ASD (17) ASD+ADHD (13)	EG: 30 (26 M, 4 F) CG: - Age: 7–16 y/o	Enhance social skills.	NEPSY-II, Triangles (Social Attribution Task).	Improvements in emotion recognition, social attribution, and executive function.
Ip et al. [29] 2016	ASD or suspected ASD	EG: 52 CG: - Age: 6–11 y/o	Enhance emotional and social adaptation skills.	FT, ET, PEP-3.	Emotion recognition: SD in ET. Affective expression: SD. Social reciprocity: SD. Overall SD for PEP-3.
Lorenzo et al. [30] 2016	ASD	EG: 20 (14 M, 6 F) CG: 20 (15 M, 5 F) Age: 7–12 y/o	Improve emotional skills.	Specific emotional script. Computer vision system to obtain child’s expressions.	Significant improvement in emotional competences.
Wade et al. [31] 2016	ASD	EG: 20 (19 M, 1 F) CG: - Age: 13–18 y/o	Develop daily living skills (driving).	Physiological and EEG data. Gaze data. Subjective observations.	The system may be beneficial in teaching driving skills. SD in most of the measures.
Ke and Lee [32] 2015	HFASD	EG: 3 CG: - Age: 8–11 y/o	Social skills development.	Qualitative time-series and micro-behavior analyses.	Practice and develop flexibility, identity, and norm construction.
Chen et al. [33] 2015	ASD	EG: 3 (2 M, 1 F) CG: - Age: 10–13 y/o	Identify the 6 core emotions.	Correct assessment rates.	SD for all participants.
Cheng et al [34] 2015	ASD	EG: 3 (3 M) CG: - Age: 10–12 y/o	Improve social understanding and skills.	2 specific scales: Social events card and social behaviors scale	Improvement in the utilization of reciprocal interactions.
Kim et al. [35] 2015	HFASD	EG: 19 (13 M, 6 F) CG: 23 (16 M, 7 F) Age: 8–16 y/o	Examining approach and tendencies in the recognition of emotions.	The final joystick position. Test for symptomatology, cognition and emotion.	EG displayed significantly less approach behavior to positive expressions to happiness than CG.
Parsons [36] 2015	ASD	EG: 6 CG: 8 Age: 7–13 y/o	Collaboration and reciprocity in behavior and communication.	Analysis of collaborative and non-collaborative interactions.	ASD children showed efforts in collaboration and reciprocity of communication.
Bai et al. [37] 2015	ASD or Asperger Syndrome	EG: 12 (10 M, 2 F) CG: - Age: 4–7 y/o	Representation of pretense and promote pretend play.	Video analysis of play behavior. Parent and participant questionnaire.	Positive effects of elicited pretend play in children with ASD.
Bekele et al. [38] 2014	ASD	EG: 10 (ASD) CG: 10 (TD) Age: 13–17 y/o	Performance in facial affect recognition.Gaze patterns.	Accuracy, response latency, and ratings of response confidence.Time spent looking at locations.	Similar accuracy at facial recognition. ASD children endorsed lower confidence, and substantial variation in gaze patterns.
Escobedo et al. [39] 2014	LFASD	EG: 12 CG: - Age: 3–8 y/o	Train selective attention.Elicitation of positive emotions.	System registration of selective and sustained attention, ability to attend the therapy, emotions.	Application seems to increase attention and improve elicitation of positive emotions.
Finkelstein et al. [40] 2014	ASD	EG: 10 CG: - Age: 8–20 y/o	Improve physical activity and motivation.	Post-experimental questionnaire. Physiological measures.	Children showed vigorous play activity and motivation to repeat the game.
Maskey et al. [41] 2014	ASD with phobia/fear	EG: 9 (9 M)CG: - Age: 7–13 y/o	Reduction of specific phobia or fear.	SCAS-P and SCAS-C, confident ratings, report of the family, anxiety report and test.	CBT techniques combined with VRE were effective in the treatment of phobia/fear in children with ASD.
Stitcher et al. [42] 2014	ASD	EG: 11 (11 M) CG: - Age: 11–14 y/o	Enhance social competence in ASD.	SRS, BRIEF, RMET, Faux Pas Stories, Strange Stories, DANVA-2-CF; D-KEFS; CPT-II.	Improvement in social responsiveness and executive functioning skills.
Bekele et al. [43] 2013	HFASD	EG: 10 (8 M, 2 F) CG: 10 (8 M, 2 F) Age: 13–17 y/o	Evaluate usability.Behavioral and physiological difference.	Performance data, eye tracking indices and physiological features.	Differences in the way adolescents with ASD process and recognize emotional faces compared to their TD peers.
Bernardini et al. [44] 2013	ASD	EG: 19 CG: - Age not specified	Acquire social communication skills in ASD.	Assessment based on a structured table-top turn-taking activity (social skills).	Game seems to improve few aspects of social skills.
Cai et al. [45] 2013	ASD	EG: 15 (13 M, 2 F) CG: - Age: 6–17 y/o	Intervention in nonverbal gesturing communication.	TONI-3 and GARS tests. Observation of final task (dolphin training).	Inconclusive data, no statistical analysis.
Fengfeng Ke & Tami Im [46] 2013	HFASD or Asperger Syndrome	EG: 4 (2 M, 2 F) CG: - Age: 9–10 y/o	Improve social interaction.	Physical and virtual communication behaviors, SSQ, Perception of Emotion.	Improvement in performance of social tasks after VR intervention.
Lorenzo et al. [47] 2013	Asperger Syndrome	EG: 20 (16 M, 4 F) CG: - Age: 8–15 y/o	Improve social skills and executive functions.	Interviews (teachers) and assessment of behavior during tasks.	Improvement of executive functions and social skills. Some skills were transferred to school context.
Modugumudi et al. [48] 2013	ASD	EG: 10 (9 M, 1 F) CG: 10 (M) Age: 7–19 y/o	Recognition and expression of emotions.	Neurophysiological measures pre- & post-treatment: EEG, EOG.	Significant improvement in children with CVE intervention program.
Wang & Reid [49] 2013	ASD	EG: 4 (3 M, 1 F) CG: - Age: 6–8 y/o	Train contextual processing of objects.	FIST-m, ASS, VR test of contextual processing of objects, final feedback questionnaire.	Improvement in contextual processing of objects and cognitive flexibility.
Alcorn et al. [50] 2011	ASD	EG: 32 (29 M, 3 F) CG: - Age: 5–14 y/o	Teach children to follow a virtual character’s gaze and gesture cues.	Observational and video data. Reaction time.	Children were able to successfully complete the tasks. Perception of the VR character as an intentional being.
Milne et al. [51] 2010	HFASD or Asperger Syndrome	EG: 14 Age: 6–15 y/o	Social skills.	Pre-test and post-test questions in each round.	Children gained information about conversation and bullying skills.

ABAS-II: Adaptive Behavior Assessment System, second edition; ADHD: Attention Deficit Hyperactivity Disorder; ASS: Attention Sustained Subtest; BRIEFSR: Behavior Rating Inventory of Executive Function-Self Reported; CG: control group; CBT: Cognitive-Behavioral Therapy; CPT-II: Conner’s continuous Performance Test-II; CVE: Collaborative Virtual Environment; DANVA-2-CF: The Diagnostic Analysis of Non-Verbal Accuracy-2, Child Facial expressions; D-KEFS: Delis–Kaplan Executive Functioning System; EEG: Electroencephalography; EG: Experimental Group; EOG: Electrooculography; ET: Eyes Test; F: Female; FIST-m: Flexible Item Selection Task (modified); FT: Faces Test; GARS: Gilliam Autism Rating Scale; h: hour(s); HFASD: High-Functioning Autism Spectrum Disorder; LFASD: Low-Functioning Autism Spectrum Disorder; M: Male; min: minute(s); NEPSY-II: Developmental Neuropsychological Assessment Second Edition; PEP-3: Psychoeducational Profile, third edition; sec: second(s); RMET: Reading the Mind in the Eyes Test; SD: Significant Difference; sess: session(s); SRS: Social Responsiveness Scale; SSQ: Social Skills Questionnaire; TD: Typically Developing; TOGSS: a performance-based evaluation to assess a shopping task; TONI-3: Test of Nonverbal Intelligence-Third Edition; VRE: Virtual Reality Environment; wk: week(s); y/o: years old.
